# The Tetraspanin CD53 Modulates Responses from Activating NK Cell Receptors, Promoting LFA-1 Activation and Dampening NK Cell Effector Functions

**DOI:** 10.1371/journal.pone.0097844

**Published:** 2014-05-15

**Authors:** Izabela Todros-Dawda, Lise Kveberg, John T. Vaage, Marit Inngjerdingen

**Affiliations:** Department of Immunology, Oslo University Hospital, Rikshospitalet, and University of Oslo, Oslo, Norway; Centre de Recherche Public de la Santé (CRP-Santé), Luxembourg

## Abstract

NK cells express several tetraspanin proteins, which differentially modulate NK cell activities. The tetraspanin CD53 is expressed by all resting NK cells and was previously shown to decrease NK cell cytotoxicity upon ligation. Here, we show that CD53 ligation reduced degranulation of rat NK cells in response to tumour target cells, evoked redirected inhibition of killing of Fc-bearing targets, and reduced the IFN-γ response induced by plate-bound antibodies towards several activating NK cell receptors (Ly49s3, NKR-P1A, and NKp46). CD53 induced activation of the β2 integrin LFA-1, which was further enhanced upon co-stimulation with activating NK cell receptors. Concordant with a role for CD53 in increasing NK cell adhesiveness, CD53 ligation induced a strong homotypic adhesion between NK cells. Further, the proliferative capacity of NK cells to a suboptimal dose of IL-2 was enhanced by CD53 ligation. Taken together, these data suggest that CD53 may shift NK cell responses from effector functions towards a proliferation phase.

## Introduction

NK cells are important contributors to the early immune defence against infected or transformed cells. NK cell effector functions are controlled by numerous NK cell receptors with both activating and inhibitory functions, including the human killer Ig-like receptors (KIR), the rodent C-type lectin-like Ly49 receptors, and the CD94/NKG2 and NKR-P1 receptors [1], [2]. In addition, NK cells broadly express the activating receptor NKG2D, and members of the natural cytotoxicity receptors, such as NKp46. Cytotoxicity and cytokine production induced by NK cell receptors are further regulated by integrins and co-receptors. The β2 integrin lymphocyte function-associated antigen-1 (LFA-1) is critically important for adhesion to target cells [3], and members of the signalling lymphocytic activation molecule (SLAM) and CD2 receptor families regulate NK cell activities through homotypic or heterotypic interactions between NK cells and other leukocytes [4], [5]. In NK cells, LFA-1 is activated upon interaction with its ligand intercellular adhesion molecule (ICAM) -1, and its activity is further enhanced by inside-out signals derived from engagement of activating NK cell receptors akin to T cell receptor mediated activation of LFA-1 [3].

Tetraspanins, four-transmembrane spanning domain proteins, represent yet another family of membrane proteins that may regulate cellular responses of NK cells. They consist of one large and one small extracellular loop, and two short intracellular tails. Tetraspanins modulate several fundamental cellular processes such as adhesion, motility, membrane fusion, and proliferation [6]. It has been difficult to clearly define natural endogenous ligands for tetraspanins, and most functional studies rely on artificial antibody ligation. One exception is CD81, which interacts with the hepatitis C virus envelope protein E2 [7]. A unique feature of tetraspanins is their ability to facilitate lateral associations with other cell surface molecules in so-called tetraspanin-enriched microdomains distinct from lipid rafts [6]. These interactions are mediated by the large extracellular loop, while the cytoplasmic tails link tetraspanins to the cytoskeleton and intracellular signalling molecules. In this manner, tetraspanins provide a framework for membrane proteins and intracellular signalling molecules from where distinct cellular responses may be co-ordinated [8]. Amongst molecules described to interact with tetraspanins are immune co-receptors (CD2, CD4, CD8, CD19), MHC class I and II, and integrins such as LFA-1, VLA-4 (a4β1), and aIIβ3 [6], [9]–[11]. In a process partly dependent on integrins, tetraspanins mediate both homotypic and heterotypic cell-cell interactions amongst leukocytes [12], [13].

NK cells express several tetraspanins, including CD9, CD53, CD63, CD81, CD82, and CD151. Of these, CD81 is the best characterized so far, and has been shown to negatively affect NK cell cytotoxicity and cytokine release mediated by CD16 [14], but to promote NK cell chemotaxis [15]. Reduction in NK cell cytotoxicity has also been reported upon ligation of CD82 and CD53 [16], [17], suggesting that these tetraspanins may function to dampen NK cell effector functions. While many tetraspanins are ubiquitously expressed by both immune and non-immune tissues, CD53 expression is restricted to cells of myeloid and lymphoid origin and is expressed by all mature leukocytes. Its function is still incompletely understood, but its ability to induce homotypic adhesion between leukocytes [18], [19], and its reported association with CD2 in NK cells [17], suggests it may play a role in NK cell adhesiveness.

To induce CD53-mediated responses in NK cells, we performed antibody ligation of CD53. This approach induced robust homotypic clustering of NK cells, which is characteristic for tetraspanin activation. CD53 ligation also promoted NK cell proliferative activity. Co-ligation of CD53 and activating rat NK cell receptors (Ly49s3, NKR-P1A, NKp46, and NKG2D) led to down-modulation of interferon gamma (IFN-γ) production. CD53 ligation also reduced NK cell degranulation, whilst enhancing LFA-1 activity induced by activating NK cell receptors. The data suggest that CD53 propagate signals that dampen effector functions and provide a milieu necessary for proliferation.

## Materials and Methods

### Animals and ethical considerations

Eight to 12-week-old PVG-*RT7^b^* (PVG.7B) rats were used. The PVG.7B strain expresses the CD45 allotype (RT7.2), but is otherwise used interchangeably with the standard PVG strain (RT7.1). The rats have been maintained at the Department of Comparative Medicine, Institute of Basic Medical Sciences, University of Oslo for more than 20 generations. The Department of Comparative Medicines institutional veterinarian has established the rules for feeding, monitoring, handling, and sacrifice of animals in compliance with regulations set by the Ministry of Agriculture of Norway and “The European Convention for the Protection of Vertebrate Animals used for Experimental and other Scientific Purposes”. The institutional veterinarian has delegated authority from the Norwegian Animal Research Authority (NARA). The laboratory animal facilities are subject to a routine health-monitoring program and tested for infectious organisms according to a modification of Federation of European Laboratory Animal Science Associations (FELASA) recommendations. The use of animals for this study was approved by the Norwegian Animal Research Authority (NARA), license number 12.4196. Rats were sacrified by asphyxiation with CO2 in a chamber that allows controlled input of gas, such as to reduce suffering of the animals. After asphyxiation, the neck was routinely dislocated to ascertain the death of the animals prior to dissection.

### Antibodies and reagents

Antibodies used were anti-CD3 (G4.18-FITC), anti-NKR-P1A (10/78-PE), and Streptavidin-PerCP from BD Biosciences (Franklin Lakes, NJ); anti-Vav (D-7, sc-8039), anti-PI-3 Kinase p85α (Z-8, sc-423), anti-Syk (C-20, sc-929), and anti- protein kinase C theta (PKC-θ; C-18, sc-212) from Santa Cruz Biotechnology (Santa Cruz, CA). mAbs to NKR-P1A (clone 3.2.3, mouse IgG1), Ly49s3/i3/s4/i4 (clone DAR13, mouse IgG1 [20], [21]), NKp46 (clone Wen23, mouse IgG1, a gift from Dr. E. Dissen, University of Oslo), CD53 (clone OX44, mouse IgG1), CD2 (clone OX34, mouse IgG1), anti-phosphotyrosine (clone 4G10, mouse IgG2b), and isotype-matched control mouse IgG1 (clone TIB-96, anti-Igh-5b) were purified from hybridomas in our laboratory. The NKG2D antibody was a generous gift from Dr. S. Krams [22]. Rat recombinant interleukin 2 (IL-2) was obtained from dialyzed cell culture supernatants from a CHO cell line stably transfected with a rat IL-2 expression construct, and rat recombinant IL-12 was from BioSource (Invitrogen).

### Cells and cell cultures

**Figure 1:**
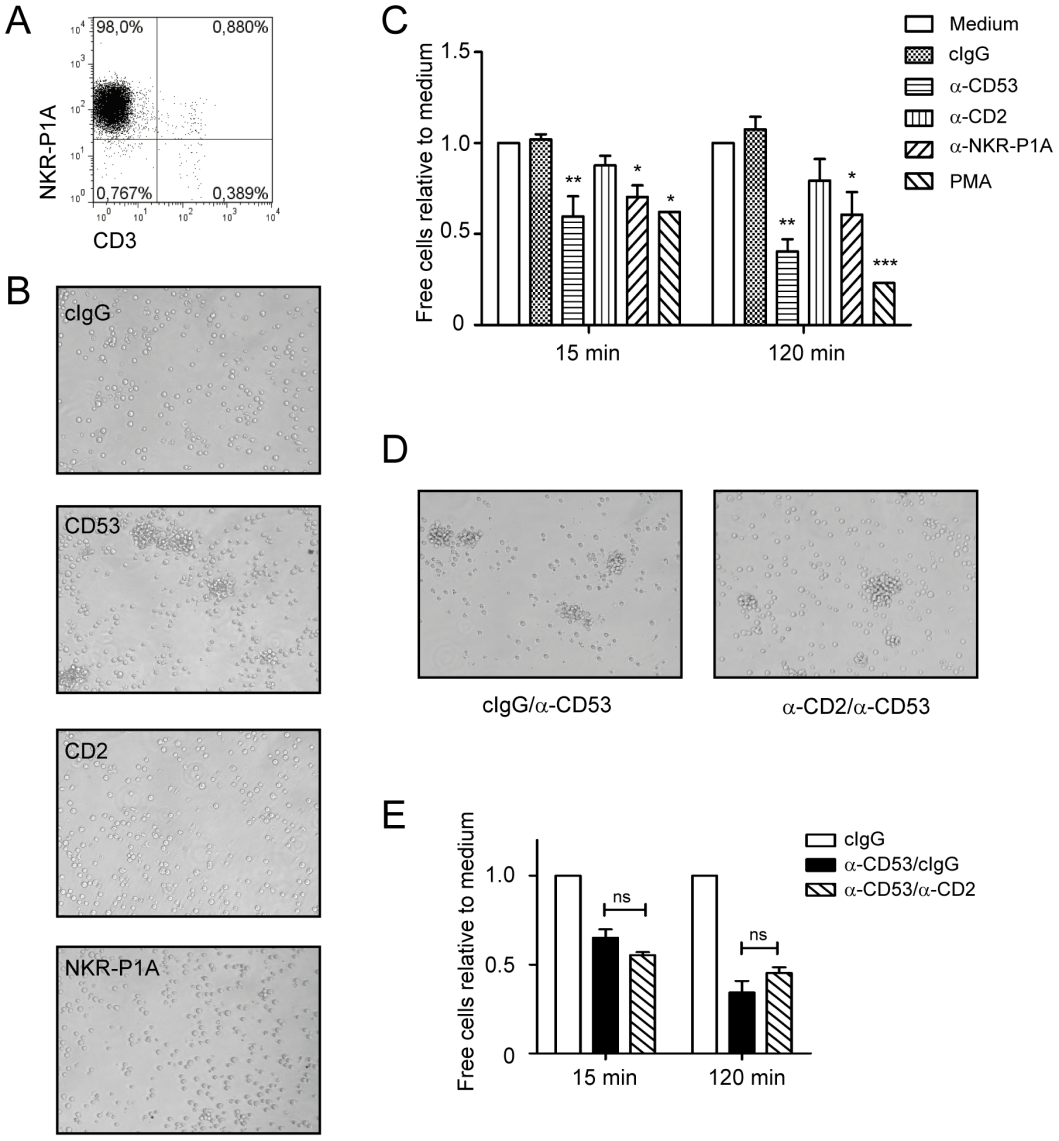
CD53 induces CD2-independent homotypic clustering of NK cells. A) Purity of 12 day-old LAK cells, as determined by staining with anti-CD3 and anti-NKR-P1A. LAK cells were cultured for 2 h in the presence of soluble antibodies towards CD53, CD2, NKR-P1A, or an isotype control IgG. B) Cluster formation was photographed with a light microscope, or C) quantified by counting the number of free, non-aggregated cells in treated samples relative to cells cultured in medium using a hemocytometer. D and E) LAK cells were co-cultured for up to 2 h with antibodies towards CD53 and CD2 or with CD53 and isotype control antibody. Cluster formation was assessed qualitatively by microscopy (D), or quantitatively by hemocytometer (E) as described above. Results are presented as the mean±SEM of 3 independent experiments with samples in duplicates (n = 6). Comparisons within an experimental group were performed with the One-way analysis of variance (ANOVA) and a *post hoc* Tukey's multiple comparisons test to compare treated samples to untreated sample. *, p<0.05; **, p<0.005; ***, p<0.001.

Mononuclear cells from spleen were prepared by Lymphoprep separation for 20 min at 650 g. Myeloid cells and B cells were depleted from the mononuclear cell suspension by incubation with nylon wool for 45 min at 37°C. Lymphokine activated killers (LAK) cells were generated by positive selection of NKR-P1A^+^ lymphocytes from mononuclear spleen cells (3.2.3-coated Pan Mouse IgG Dynabeads, Invitrogen) and culturing for >12 days in complete RPMI (cRPMI; RPMI 1640 supplemented with 10% FBS, 1 mM sodium pyruvate, 5×10^−5^ M 2-ME, and antibiotics) supplemented with IL-2. This protocol routinely yields >90% NKR-P1A^+^CD3^−^ NK cells (Fig. 1A). The rat leukemic NK cell line RNK-16 [23], the Fc^−^ mouse T cell lymphoma cell line YAC-1 (ATCC TIB-160, [24]), and the Fc^+^ mouse macrophage cell line P388D1 (ATCC CCL-46, [25]) were maintained in cRPMI.

### CD107a degranulation assay and ^51^Cr release

Degranulation was measured using a hamster anti-rat CD107a antibody generated in our laboratory (unpublished). Nylon wool non-adherent lymphocytes (1×10^6^ cells/ml) were mixed with YAC-1 target cells (1×10^6^ cells/ml) at 1∶1 ratio in 96-well plates, spun and incubated in the presence of anti-CD107a-FITC and 10 µg/ml of anti-CD53 or isotype control antibody in cRPMI for 4 h. GolgiStop (BD Biosciences) was added after 1 h of incubation. Cells were then surface stained and fixed in 2% paraformaldehyde prior to FACS analysis in PBS supplemented with 0.5% FBS and 2 mM EDTA. Cytotoxicity was measured with a standard ^51^Cr release assay, using LAK cells or RNK-16 cells as effector cells and ^51^Cr-labelled YAC-1 or P388D1 cells as target cells as previously described [26]. Anti-CD53 or isotype control antibodies were added to LAK or RNK-16 cells at 10 µg/ml 15 min prior to addition of target cells in the indicated effector to target (E∶T) ratios in triplicates. Spontaneous release was below 5% of the total cpm of the target cells. Results are presented as mean values from triplicates for each E∶T cell ratio, error bars representing standard deviation from one experiment out of three separate, representative experiments.

### Intracellular IFN-γ staining

Mononuclear spleen cells were Ig-depleted using sheep anti-rat Dynabeads (Invitrogen, 70 µl beads/2×10^7^ cells), and seeded at 3×10^6^ cells/ml in 96-well plates pre-coated with 10 µg/ml of the indicated mAbs in PBS overnight at 37°C. Spleen cells stimulated with IL-12 (2 ng/ml; Invitrogen) and IL-2 were used as a positive control. Cells were incubated for 6 hrs, with addition of Brefeldin A (Sigma-Aldrich) at 5 µg/ml for the last 3 h of culture. Cells were harvested and surface stained, then fixed for 10 min in 2% paraformaldehyde, permeabilized with 0.5% saponin in PBS for 20 min, and stained with PE-conjugated anti-rat IFN-γ (BD Biosciences). NK cells were gated as NKR-P1A^+^CD3^−^ cells.

### Cell stimulation, immunoprecipitation and western blotting

Cells were stimulated using Pan Mouse IgG Dynabeads (Dynal, Invitrogen) coated with combinations of control IgG, anti-CD53 or anti-Ly49s3 mAbs as indicated in the figures (0.5 µg each mAb/25 µl beads). LAK cells were washed and resuspended in PBS at 2×10^8^ cells/ml at room temperature, and pre-incubated at 37°C for 10 min prior to stimulation with 25 µl Dynabeads/1×10^7^ LAK cells for 1 min. Cells were immediately lysed by addition of an equal volume of 2x ice-cold lysis buffer (2% Nonidet P-40, 20 mM Na_3_VO_4_, 2x protease inhibitor cocktail, 2 mM PMSF (all from Sigma), and 2x PhosSTOP phosphatase inhibitor (Roche), 300 mM NaCl and 50 mM Tris, pH 7.4), then spun at 10 K *g* for 10 min. For immunoprecipitations, Protein G Dynabeads (Dynal, Invitrogen) were pre-coated with relevant antibodies for 30 min at room temperature, prior to incubation with cell lysates for 2 h at 4°C, washed 3x in 1x lysis buffer, and resuspended in SDS sample buffer. Proteins were separated by SDS-PAGE, transferred onto PVDF membranes (Millipore), and detected with primary antibodies in either 3% BSA or 5% skimmed milk in TBS with 0.05% Tween overnight at 4°C, and for 1 h with secondary horseradish peroxidase-conjugated goat anti-mouse IgG, goat-anti mouse IgG2b, or goat anti-rabbit IgG (Jackson ImmunoResearch Laboratories). Proteins were visualized by enhanced chemiluminescence (Pierce, UK). To probe with another antibody, membranes were stripped, washed, and then re-blotted.

### Cell aggregation and conjugate assays

LAK cells were washed in RPMI medium, and adjusted to 2.5×10^6^ cells/ml in RPMI supplemented with 2% FBS and 20 mM Hepes. Antibodies were added at 10 µg/ml, and 100 µl cells were plated in 96-well plates and incubated at 37°C for the indicated times. Photographs were recorded after 2 h of incubation with a light microscope. To quantify aggregate formation, a method from Rothlein *et al* [27] was adopted, where free, non-aggregated cells were enumerated in a hemocytometer, and the number of free cells in treated samples were related to the number of free cells in samples incubated in medium alone.

For conjugate assays, RNK-16 cells were labeled with 0.1 µM carboxyfluorescein succinimidyl ester (CFSE; Invitrogen, Molecular Probes) at 1×10^7^ cells/ml in PBS and 2% FBS for 10 min at 37°C. Target cells (YAC-1) were stained with seminaphtharhodafluor (SNARF-1) (Molecular Probes, Invitrogen) according to the manufacturer's protocol. NK cells (1×10^7^ cells/ml) were incubated with either anti-CD53 mAb or isotype control mAb at 10 µg/ml for 30 min on ice. 100 µl NK cells (1×10^5^ cells) were mixed with 100 µl target cells (1×10^5^ cells) with an effector to target ratio 1∶1, spun for 30 s at 300 g, and incubated at 37°C for 10 min. Reactions were stopped by addition of 2% paraformaldehyde. Conjugate formation was analyzed by flow cytometry, and the percentage of NK cells in conjugates with target cells calculated.

### Soluble ICAM-1 binding assay

To assess binding of ICAM-1 to NK cells, recombinant rat ICAM-1/human Fc fusion protein was reacted with anti-human Fc-PE on ice, following a protocol adapted from Konstandin *et al* [28]. Briefly, anti-human Fc-PE was diluted 1∶6.25 in PBS containing 100 µg/ml rat ICAM-1/human Fc fusion protein for 30 min on ice. LAK cells or nylon wool enriched fresh NK cells were washed in PBS, preincubated with 1 µg/1×10^6^ cells of indicated antibodies on ice for 30 min, and washed with cold PBS. Cells were resuspended in 31.25 µl of PBS containing 0.5% BSA, 0.9 mM Mg^2+^, and 0.5 mM Ca^2+^. Immediately prior to the assay, cells were added 6.25 µl of ICAM-1/PE complexes, and added 12.5 µl crosslinking antibody (1 µg/1×10^6^ cells) (in PBS containing Mg^2+^/Ca^2+^ as above). As positive control, NK cells were stimulated with 100 mM Mg^2+^/10 mM EGTA in PBS supplemented with 0.5% BSA only. Cells were stimulated for 5 min then fixed with 2% paraformaldehyde. Binding of ICAM-1 was evaluated by flow cytometry.

### CFSE proliferation assay

Nylon wool non-adherent lymphocytes were labeled with 5 µM CFSE (Invitrogen, Molecular Probes) at 1×10^7^ cells/ml in PBS and 2% FBS for 10 min at 37°C. Cells were washed, and plated in 24-well plates at 1×10^6^ cells/ml in the presence of sub-optimal concentrations of IL-2 (10 times lower dose), and either control IgG or anti-CD53 at 10 µg/ml. Cells were harvested at day 7, surface stained with anti-NKR-P1A and anti-CD3, and analyzed by flow cytometry.

### Statistical analysis

Graphics and statistical analysis were performed with the GraphPad Prism software. Data are presented as the mean ± standard error of the mean (SEM). Comparisons within an experimental group were performed with the parametric One-way analysis of variance (ANOVA) in combination with a *post hoc* Tukey's multiple comparisons test to compare treated to untreated samples, or with a parametric two-sided unpaired *t* test as indicated. *P* values less than 0.05 were considered statistically significant.

## Results

### CD53 antibody ligation induces CD2-independent homotypic adhesion of NK cells

The precise mechanism by which tetraspanins are activated is unclear, and most studies of this protein family rely on antibody ligation in order to induce responses. Such ligation of tetraspanins has previously been shown to induce homotypic aggregation of B cells and thymocytes [13], [18], [19]. The same approach was used to examine CD53-mediated responses in rat NK cells. CD53 antibody ligation (using the monoclonal OX44 antibody) of highly pure NKR-P1A^+^CD3^−^ LAK cells (Fig. 1A) induced robust homotypic clustering of NK cells within 2 h at 37°C (Fig. 1B), which is characteristic for tetraspanin activation. In contrast, no such aggregates were observed upon ligation of the strong activating rat NK cell receptor NKR-P1A or isotype-matched control antibodies (Fig. 1B). This shows that the formation of aggregates is specifically induced upon antibody binding to the tetraspanin and not secondary to Fc receptor binding. Cluster formation was also quantified by counting free cells in the samples related to cells incubated in medium alone. We observed clustering of NK cells as early as 15 min after addition of CD53 antibodies, and the clustering kinetics was comparable to that induced by the positive control phorbol 12-myristate 13-acetate (PMA) (Fig. 1C). There were also reductions of free cells in the presence of NKR-P1A antibodies, and although we did not observe large aggregates in the presence of NKR-P1A by microscopy, it is possible that several smaller aggregates are formed in contrast to the large clusters formed by CD53 or PMA (not shown).

CD53 has been shown to interact with CD2 [17], which is expressed by the majority of NK cells. In rodents CD2 binds CD48, another member of the CD2 family of receptors expressed by NK cells, and this receptor pair enables homotypic adhesion between NK cells. To test whether CD53-induced homotypic adhesion was dependent on CD2, we introduced an anti-CD2 antibody (OX34) that prevents binding to CD48 [29]. Although the OX34 antibody is capable of stimulating NK cells upon crosslinking [30], the antibody on its own did not induce homotypic NK cell clustering (Fig. 1B and C). Abrogating the putative interaction between CD2 and CD48 did not affect homotypic adhesion mediated by CD53 (Fig. 1D and E), demonstrating that CD53-mediated adhesion occurs independently of CD2.

### Increased adhesiveness induced by CD53 is partly mediated by LFA-1 activation

**Figure 2:**
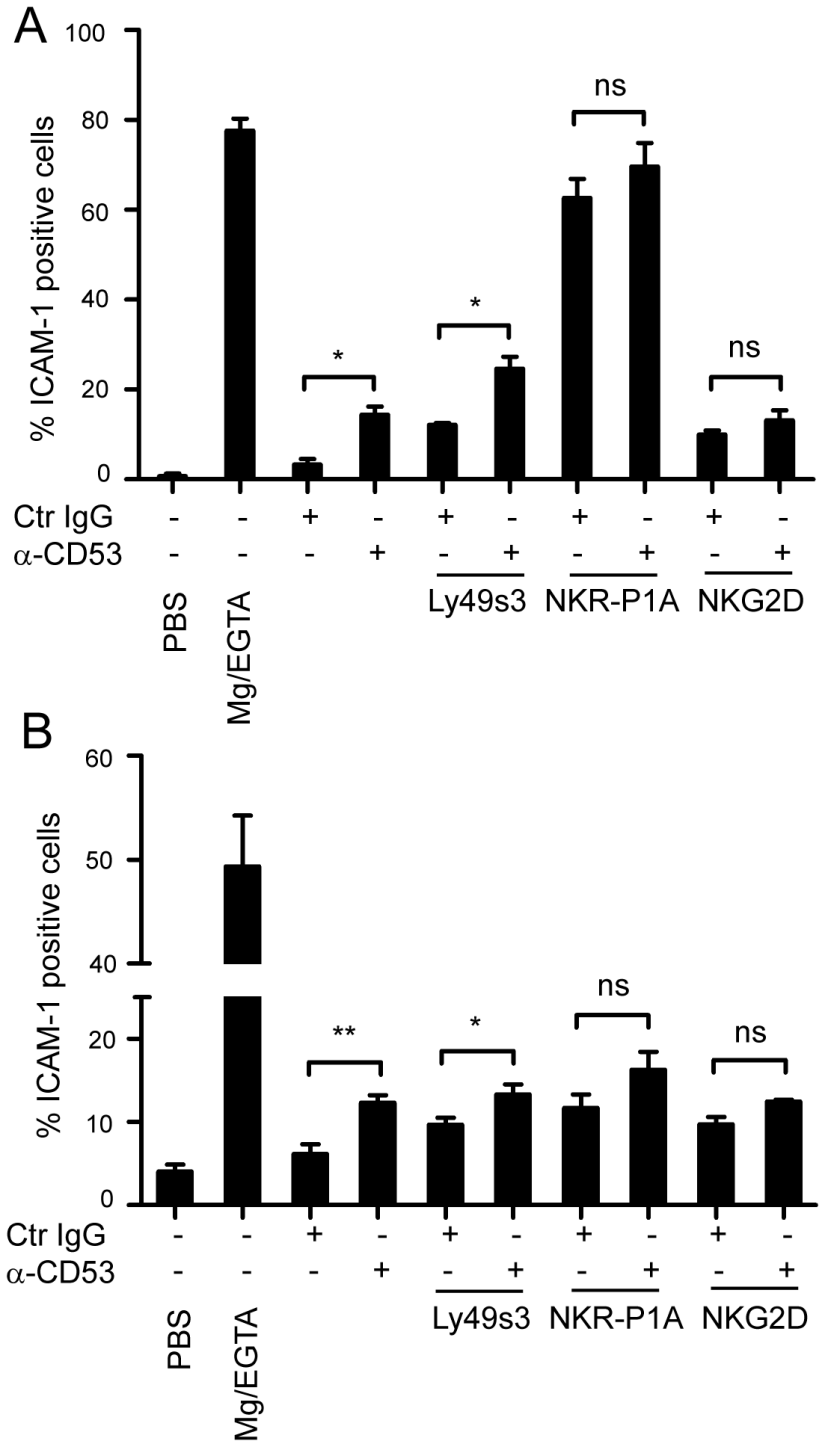
Elevated LFA-1 activation in response to CD53 ligation, but normal target conjugation and reduced migratory response. A) Enriched, primary NK cells, or B) LAK cells were pre-incubated with the indicated antibodies for 30 min on ice. Stimulation was induced for 5 min at 37°C in PBS supplemented with Ca^2+^ and Mg^2+^ with a crosslinking secondary F(ab)_2_ anti-mouse antibody in the presence of a soluble rat ICAM-1/Fc-fusion protein. PBS with Mg^2+^/EGTA was used as positive control. Binding of ICAM-1 to NKR-P1A^+^CD3^−^ NK cells was assessed by flow cytometry. The data represents 3–5 independent experiments with samples in duplicates (n = 6–10), and are presented as the mean±SEM. Data were analysed using the two-tailed unpaired t-test. *, p<0.05; **, p<0.005.

Several studies have shown that tetraspanin-mediated homotypic adhesion between T cells or B cells are mediated in part by their activation of the β2 integrin LFA-1 [12], [13], [18], [19]. To address whether LFA-1 is activated in response to CD53 ligation in NK cells, cells were pre-incubated with CD53 antibodies, and LFA-1 activity was assessed by measuring binding of a soluble ICAM-1 Fc fusion protein. CD53 ligation induced LFA-1 activation in both primary (Fig. 2A) and IL-2 cultured NK cells (Fig. 2B), indicating that CD53 can directly enhance NK cell adhesion through LFA-1. In addition, co-ligation of CD53 together with NKR-P1A, Ly49s3 (one of the most abundantly expressed activating Ly49 receptor in the PVG strain rat [31]), or NKG2D showed that CD53 synergistically enhance LFA-1 activation induced through Ly49s3 of both primary and IL-2-cultured NK cells. Increased LFA-1 activity was also observed upon co-ligation of NKR-P1A and NKG2D with CD53, although it did not reach a statistical difference from activity induced by either of these activating receptors alone. Of note, NKR-P1A-induced LFA-1 activation was very potent, almost reaching the levels induced by the positive control Mg^2+^ and EGTA on primary cells. Although LFA-1 activity may certainly contribute to the observed homotypic aggregates induced by CD53 ligation, other mechanisms must also be operating as only CD53 and not NKR-P1A ligation caused cell clustering (Fig. 2A).

### CD53 ligation induces decreased NK cell responses in response to tumor targets or activating NK cell receptor stimulation

**Figure 3:**
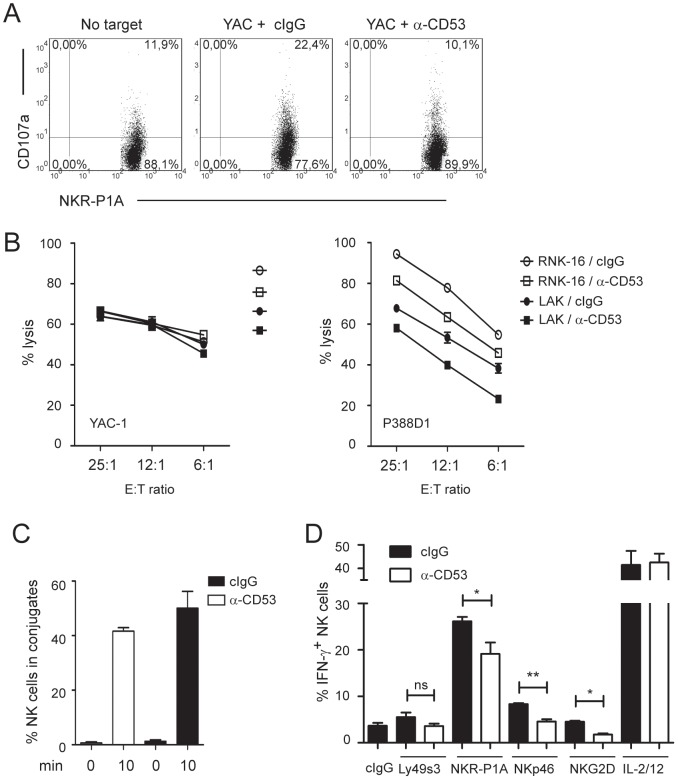
CD53 ligation reduces NK cell degranulation and receptor-induced IFN-γ production. A) Degranulation of primary NKR-P1A^+^CD3^−^ NK cells in response to YAC-1 target cells was assessed by flow cytometry. Lymphocytes and YAC-1 cells mixed 1∶1 were co-cultured for 4 h with anti-CD107a antibodies in combination with anti-CD53 or isotype control antibodies. B) Cytotoxicity of RNK-16 cells (open symbols) or LAK cells (filled symbols) against Fc^−^ YAC-1 target cells (left panel) or Fc^+^ P388D1 target cells (right panel) in the presence of anti-CD53 or isotype control antibodies were determined with a standard 4 h ^51^Cr release assay. Data represent the mean values of triplicates ± SEM from one representative experiment out of three. C) Conjugate formation between CFSE-stained RNK-16 cells and SNARF-1 stained YAC-1 target cells was assessed by flow cytometry. Percent NK cells in conjugates with target cells was calculated as described in Materials and methods. The data are representative of 3 independent experiments, and presented as the mean±SEM. D) Ig-depleted spleen cells were stimulated for 6 h *in vitro* by the indicated plate-bound antibodies or cytokines, and the proportion of IFN-γ positive cells was assessed by flow cytometry gating on NKR-P1A^+^CD3^−^ NK cells. The data represents 3 independent experiments (n = 3), and are presented as the mean ± SEM. n = 3. Data were analysed using the two-tailed unpaired t-test. *, p<0.05; **, p<0.005.

CD53 ligation was previously shown to induce phosphoinositide turnover and calcium flux in the rat NK cell line RNK-16, but also to reduce killing of YAC-1 target cells suggesting that CD53 modulates NK cell effector responses [17]. Concordantly, we show here that degranulation of splenic NK cells in response to YAC-1 target cells was reduced upon CD53 ligation (Fig. 3A). Degranulation was assessed by measuring deposition of the cytotoxic granule marker CD107a on the cell surface by flow cytometry. By contrast, there was no significant effect of CD53 ligation on cytotoxicity towards YAC-1 target cells using either freshly isolated NK cells (data not shown), LAK cells, or RNK-16 cells (Fig. 3B, left panel). However, the CD53 antibody evoked a weak redirected inhibition of cytotoxicity towards the Fc-receptor^+^ NK sensitive tumor target P388D1 (Fig 3B, right panel), indicating that CD53 transduce signals that may negatively affect NK cytotoxicity. In contrast, conjugate formation between RNK-16 cells and YAC-1 target cells was unperturbed upon ligation of CD53 (Fig. 3C). If anything there was an increase in conjugates which would be in line with the pro-adhesive effects of CD53 ligation. Thus, although CD53 ligation decreases degranulation and evoke redirected inhibition of cytotoxicity, conjugate formation and adhesion appears to be in the normal range.

We next assessed whether IFN-γ production, the other major NK cell effector function, was affected upon CD53 ligation. Freshly isolated spleen cells were stimulated with plate-bound antibodies towards the activating receptors NKR-P1A, Ly49s3, NKp46, or NKG2D in combination with either anti-CD53 or isotype control antibodies. NK cells produced reduced levels of IFN-γ upon co-ligation of CD53 and activating receptors compared to ligation of each activating receptor alone (Fig. 3D). Of note, NKR-P1A is a particularly strong activation receptor, inducing a brisk IFN-γ response that was partly inhibited by CD53 stimulation. The same was the case for the weak responses mediated by NKp46 or Ly49s3, while the NKG2D receptor failed to induce IFN-γ levels above that of isotype antibodies alone. The stronger IFN-γ response induced by IL-2 and IL-12 was not inhibited by CD53. Collectively, these findings show that CD53 reduces both degranulation and IFN-γ production induced by several activating receptors, suggesting it may function to dampen NK cell activity.

### IL-2-dependent NK cell proliferation is enhanced by CD53 ligation

**Figure 4:**
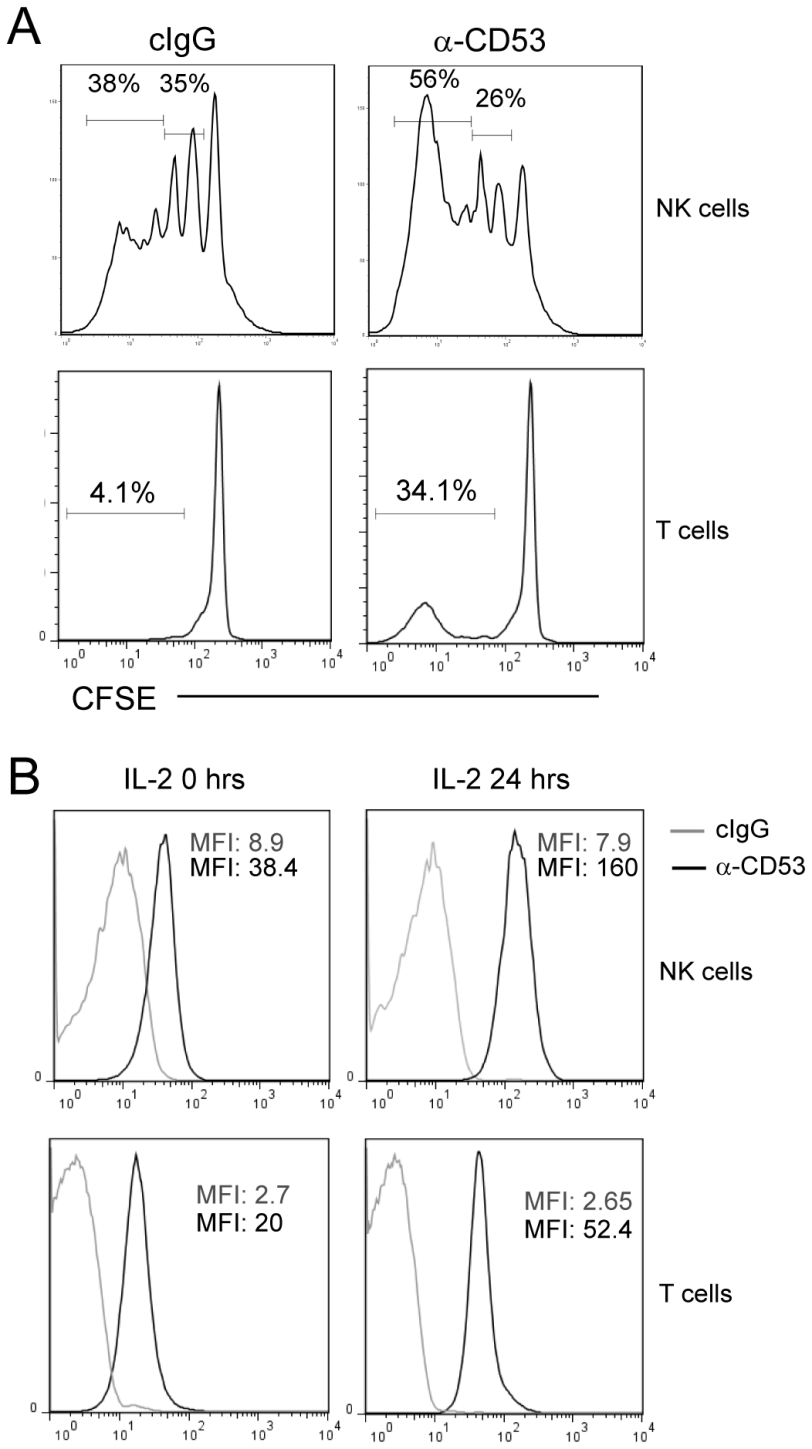
Increased proliferative activity of NK cells in response to CD53 ligation. A) CFSE-labeled lymphocytes were cultured for 7 days with a sub-optimal dose of IL-2 in the presence of an anti-CD53 antibody or isotype control antibody at 10 µg/ml. The CFSE dilution profiles of NKR-P1A^+^CD3^−^ NK cells (upper panels) or CD3^+^NKR-P1A^−^ T cells (lower panels) were analyzed by flow cytometry. Data are representative of 3 independent experiments (n = 3). B) Flow cytometric analysis of CD53 expression by primary NK cells (upper panels) or T cells (lower panels) before and after culture in IL-2 for 24 h. NK cells were gated as NKR-P1A^+^CD3^−^ cells. Shown are stainings with anti-CD53 (solid line) and an isotype control antibody (grey line). n = 3.

Homotypic and heterotypic clustering of T cells and NK cells is suggested to provide co-stimulatory signals that may enhance both effector functions as well as proliferative activity. To test the effect of CD53 ligation on NK cell proliferation, freshly isolated spleen lymphocytes (depleted of monocytes and B cells) were cultured in sub-optimal doses of IL-2 in a 7-day CFSE proliferation assay. Increased proliferative activity of NK cells was detected upon co-culture with CD53 antibodies, as compared to culturing with isotype control antibodies (Fig. 4A). This suggests that CD53 may potentiate the proliferative activity of NK cells. Also, a fraction of T cells divided upon culture with sub-optimal IL-2 and CD53 antibodies, in contrast to IL-2 alone (Fig. 4A). Further, the expression levels of CD53 were enhanced on both NK cells and T cells upon IL-2 activation, detectable already after 24 h of culture (Fig. 4B), which could indicate that IL-2 may further potentiate clustering induced by CD53.

### CD53 co-stimulation alter early proximal signalling from Ly49s3

**Figure 5:**
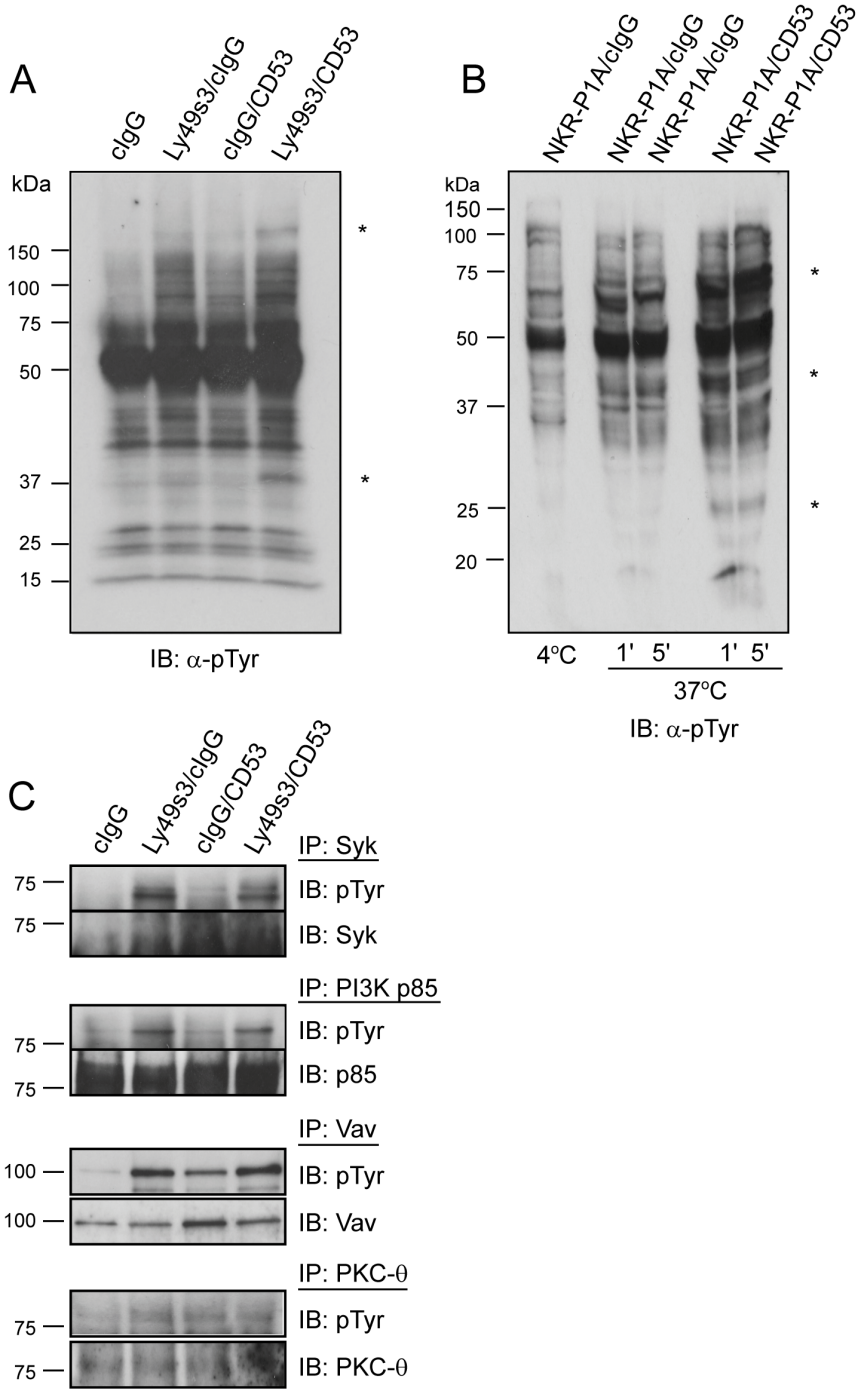
CD53 enhances protein tyrosine phosphorylation induced by activating NK receptors, but not of the most proximal signalling molecules. A) LAK cells were stimulated with Dynabeads co-coated with combinations of anti-Ly49s3, anti-CD53 or control antibodies for 1 min as indicated. Cell lysates were subjected to immunoblotting with an anti-phosphotyrosine antibody, and show enhanced tyrosine phosphorylation of bands at ∼40 and 160 kDa ∼in sampled co-ligated with CD53 and Ly49s3 antibodies. B) NKR-P1A^+^ spleen cells were positively selected with NKR-P1A-coated Dynabeads on ice, and stimulation was induced at 37°C in the presence of either anti-CD53 or isotype control antibodies for 1 or 5 min. Cell lysates were subjected to immunoblotting with anti-phosphotyrosine. The western blots are representative for 3 independent experiments, and show enhanced tyrosine phosphorylation of bands at ∼25, 40, and 75 kDa in samples co-ligated with CD53 and NKR-P1A antibodies. C) LAK cells were stimulated with Dynabeads co-coated with combinations of anti-Ly49s3, anti-CD53 or control antibodies for 1 min. Cell lysates were immunoprecipitated with antibodies to Syk, PI3K, Vav, or PKC-θ, then immunoblotted with either an anti-phosphotyrosine antibody or the immunoprecipitating antibody as control. The data are representative for 3 independent experiments.

Ligation of CD53 modulated both IFN-γ production and LFA-1 adhesiveness induced by activating NK cell receptors. To address whether intracellular signalling pathways induced by activating receptors are modulated by CD53, we co-ligated LAK cells with antibodies towards CD53 and Ly49s3. Ly49s3 is expressed by the majority of LAK cells after 10-12 days of culture [20]. We detected enhanced tyrosine phosphorylation of proteins at ∼160 kDa and ∼40 kDa after co-ligation with Ly49s3 and CD53 antibodies for 1 min, compared to co-ligation with anti-Ly49s3 and isotype control antibodies (Fig. 5A). These two proteins were not tyrosine phosphorylated by CD53 or Ly49s3 alone. Similarly, enhanced tyrosine phosphorylation of bands at ∼25 kDa, ∼40 kDa and ∼75 kDa were detected upon co-ligation of NKR-P1A and CD53 on freshly isolated NK cells upon 1 or 5 min of stimulation, compared to NKR-P1A and isotype control antibody stimulation (Fig. 5B).

To determine the identity of the differentially phosphorylated proteins, we performed immunoprecipitations of several proteins known to act in the signalling pathways downstream of activating NK cell receptors, such as Syk, phosphoinositide 3-kinase (PI3K), phospholipase C gamma (PLC-γ), Vav, and PKC-θ. Again LAK cells were stimulated for 1 min with Dynabeads co-coated with anti-Ly49s3 and anti-CD53, anti-Ly49s3 and control IgG, anti-CD53 and control IgG, or control IgG alone. Ligation of CD53 led to tyrosine phosphorylation of Vav and PKC- θ, but no phosphorylation of Syk, PI3K or PLC-γ (Fig. 5C, and data not shown). Stimulation with anti-Ly49s3-coated Dynabeads led to phosphorylation of all five proteins, but this phosphorylation was not affected by CD53 co-ligation. Also, phosphorylation of c-Cbl, Lck, linker of T cell activation (LAT), and Grb2 were not affected upon CD53 co-ligation with Ly49s3 (data not shown). The identity of the phosphorylated proteins detected in Figures 5A and B is thus at present unclear.

## Discussion

NK cells express several tetraspanins, among them CD53 which is expressed by all mature leukocytes. The precise functional role of CD53 on NK cells is not known, although it was previously shown to reduce NK cell lytic activity [17]. Most functional studies of tetraspanins rely on antibody ligation, as their mode of primary activation is still largely unknown. Here, we show that CD53 ligation induced LFA-1 activation and synergistically enhanced LFA-1 activation induced by activating NK cell receptors. Increased adhesiveness was accompanied by reduced IFN-γ production and degranulation, weak reductions in cytotoxicity, and enhanced proliferative activity suggesting that CD53 could play a role in promoting NK cell proliferation.

A common feature for several tetraspanins is their ability to facilitate homotypic adhesion between cells upon antibody ligation. Here, CD53 antibody ligation rapidly promoted homotypic clustering of NK cells, which has also been reported for thymocytes and B cells [18], [19]. Homotypic adhesion induced by tetraspanins is shown to partly depend on LFA-1 activation [13], [18], as it is not completely abrogated using blocking antibodies [12], [19]. The LFA-1 independent mechanism is still unclear, but most likely involves either other integrins or adhesion molecules. In our hands, CD53 induced LFA-1 activation, and although we did not attempt blocking LFA-1/ICAM-1 interactions during the homotypic adhesion assay, LFA-1 probably contributes to some extent to the observed cell clustering. CD2, which co-stimulates LFA-1 adhesion and NK cell effector functions [32], associates with CD53 in NK cells and T cells [17]. CD2 has been implicated in homotypic NK cell interactions, through interactions with either rodent CD48 or human CD58 [5], [33]. However, we found that CD53-mediated adhesion was not prevented by introducing an antibody that blocks the interaction between CD2 and CD48 (OX34; [29]), suggesting that CD53 promotes homotypic adhesion independently of CD2. It is presently unclear whether CD53 can facilitate homotypic clustering on its own, or via other yet unidentified surface molecules.

In general, homotypic clustering of lymphocytes may function to facilitate a milieu where the threshold for cellular activation is lowered. In T cells, the formation of homotypic clusters has been suggested to promote synapse-based delivery of IL-2 between T cells, which may ensure efficient initiation of T cell proliferation [34]. We show here that CD53 ligation enhance NK cell proliferation in response to IL-2, suggesting similar mechanisms may operate for NK cells. Interestingly, CD53 also induced proliferation of a subset of T cells under the same conditions, although not to the same extent as for NK cells. As the culture contained both NK cells and T cells, CD53 may facilitate the pro-proliferative response by acting on both NK cells and T cells. It could be hypothesized that the formation of clusters between T cells and NK cells and/or between T cells could possibly promote delivery of IL-2 from T cells needed for NK cell proliferation.

Increased surface levels of CD53 in response to IL-2 were detected on both NK cells and T cells. Interestingly, up-regulation of CD53 under inflammatory conditions is also observed on macrophages in response to lipopolysaccharide [35], as well as on leukocytes obtained from rheumatoid arthritis patients [36], patients with atopic eczema [37], or in lesions after spinal cord injury [38]. The up-regulation of CD53 under inflammatory conditions is suggested to protect leukocytes from apoptosis during inflammation, underpinned by studies demonstrating that ligation of CD53 protect cells from apoptosis and promote increased cell survival [35], [39]. CD53 is also implicated in survival during thymic selection [40]. Moreover, elevated levels of CD53 expression is found on different cancers, such as B-cell leukemia and lymphomas [41], [42], suggesting it may contribute to increased survival of the tumour cells.

CD53 ligation caused a dampening of IFN-γ production in NK cells in response to stimulation of activating NK cell receptors. In support of CD53 acting as a suppressor of inflammatory cytokine production, a genome-wide linkage analysis has demonstrated an association of CD53 with reduced innate production of tumor necrosis factor (TNF)-α [43], and CD53 was shown to suppress cytokine production by monocytes in an *in vitro* asthma model [44]. Thus, CD53 may serve to dampen certain inflammatory conditions.

While we observed reduced degranulation in response to YAC-1 target cells, the cytotoxic response towards YAC-1 appeared normal. While this may seem contradictory, degranulation may not always predict ability to kill. While NK cell lines and LAK cells efficiently kill diverse tumor targets, it is only possible to detect CD107a on primary, freshly isolated NK cells ([45] and Kveberg L., unpublished observation). There may also be sensitivity differences between the two assays, where a potential inhibitory input from CD53 may be undetectable in the ^51^Cr release assay, but detectable in the degranulation assay. The ability of CD53 to negatively influence target killing, was demonstrated by the induction of redirected inhibition of cytotoxicity using the Fc^+^ target cell line P388D1. This suggests that crosslinking CD53 by using target cells induce a stronger response than by antibodies alone. Unlike our study, CD53 ligation was previously shown to reduce RNK-16 mediated killing of YAC-1 targets [17]. The discrepancy between our study and the reduced cytotoxicity observed by Bell and co-workers [17] could be due to their use of another antibody clone towards CD53 (7D2) than in our study (OX44).

NK cell adhesiveness is necessary for killing, but we observed diminished degranulation in response to CD53 ligation despite increased LFA-1 activity and normal conjugate formation. A similar phenomenon has been reported for CD81, which has been found to impair cytokine production and granule secretion [14], yet to promote NK cell adhesion [15]. Previously, CD53 ligation was shown to induce phosphoinositide turnover and calcium flux in NK cells, B cells, and monocytes [17], [46], [47]. These differential cellular responses may depend on the context in which the tetraspanins are engaged, the cells they are expressed in, and the molecules they associate with. For instance, while CD81 inhibits NK cell effector functions, it promotes B cell receptor activation as a part of the CD19/CD21 complex [48]. In T cells, several tetraspanin proteins, including CD9, CD81 and CD82 are reported to associate with CD4 or CD8 and enhance T cell effector functions [49], while CD81 negatively modulates FcεRI-mediated degranulation in mast cells and suppress IgE-dependent allergic reactions [50]. Considering the number of proteins tetraspanins may interact with, the effects of antibody ligation of tetraspanins may in some instances be secondary to modifications in the tetraspanin network.

Based on the present and previous studies, the physiological function of CD53 appears to be to promote leukocyte adhesion and survival. Also, a study of humans with CD53-deficiency reported a disease phenotype indicating defects in cellular adhesion [51]. In addition to promoting adhesion on its own, CD53 ligation enhances LFA-1 activity induced by the activating receptor Ly49s3. NKR-P1A-induced binding of ICAM-1 to NK cells was not further strengthened by CD53, possibly as LFA-1 activity may be near maximum upon ligation of NKR-P1A alone. This suggests that CD53 may facilitate and possibly strengthen target cell adhesion upon interaction of activating NK cell receptors with their ligands. Activating NK cell receptors signal through Src family kinases and Syk to induce downstream signaling pathways leading to degranulation and cytokine release [3]. Co-engagement of CD53 and the activating receptors Ly49s3 or NKR-P1A resulted in enhancement of protein tyrosine phosphorylation compared to ligation of either activating receptor alone. While we were not able to identify these proteins, CD53 clearly modulate some signaling proteins induced from activating receptors. While many signaling molecules involved in the downstream signaling pathways from activating receptors were not activated by CD53 ligation alone, CD53 ligation did lead to tyrosine phosphorylation of Vav and PKC-θ. Both are important for mediating activation of LFA-1. Interestingly, CD53 has previously been shown to associate with phosphatases [52], which could be responsible for the observed inhibition of cytolytic activity and/or cytokine production. In line with human studies, NKG2D ligation on rat NK cells led to minimal NK cell activation. In contrast, NKR-P1A appears to be a very strong activating receptor on rat NK cells, inducing both high IFN-γ levels and ICAM-1 binding.

In conclusion, we have shown that antibody ligation of CD53 promotes homotypic clustering of NK cells, enhance activation of LFA-1, and enhance proliferation, while diminishing degranulation and IFN-γ production. We suggest that stimulation through CD53 may shift NK cell activities from effector functions towards preparation for proliferation. During *in vivo* immune reactions, such clustering may also encompass CD53-dependent heterotypic interactions between other immune cells that may further regulate NK cell responses. How CD53, or other tetraspanins, is naturally engaged and activated is presently unknown, but its activation could be induced upon interactions with other membrane proteins.
